# Endoscopic ultrasound-guided Hartmann reversal procedure

**DOI:** 10.1055/a-2873-8589

**Published:** 2026-06-03

**Authors:** Mateusz Jagielski, Przemysław Kasprzyk, Jacek Piątkowski, Piotr Narbutt, Łukasz Dziki, Marek Jackowski, Michał Spychalski

**Affiliations:** 1 Department of General, Gastroenterological and Oncological Surgery, Collegium Medicum 49577 Nicolaus Copernicus University in TorunTorunPoland; 2 Department of General and Oncological SurgeryBrzeziny Specialist HospitalBrzezinyPoland; 3 Department of General and Oncological Surgery 37808 Medical University of LodzLodzPoland

Despite the widely known deterioration in the quality of life in patients with a stoma, Hartmann’s procedure still constitutes an essential element in the treatment of many colorectal diseases, in both emergency and elective settings 1 . The classic surgical restoration of gastrointestinal continuity following Hartmann’s procedure is frequently a technical challenge, and the operation is associated with a high risk of complications reaching up to 25% 2 .

The innovative Endoscopic Ultrasound-guided Hartmann Reversal Procedure (EndoHARP) addresses the challenges associated with restoring gastrointestinal continuity in patients who have undergone Hartmann’s procedure. This intervention utilizes minimally invasive endoscopic techniques, allowing for safe bowel anastomosis without the laparotomy and can be considered suitable for both benign conditions, such as diverticular perforation, and malignant lesions presenting with obstruction performed following the completion of oncological treatment.

The EndoHARP procedure consists of three stages. The first stage involves a modified Hartmann’s procedure, in which the mobilized sigmoid or descending colon is laterally attached to the closed rectal stump with seromuscular sutures. The second stage, performed after a minimum of 3 months, involves the endoscopic creation of the anastomosis. Using an echoendoscope introduced transrectally, under EUS and fluoroscopic guidance, the previously fixated bowel loop is precisely punctured, and then the anastomosis is created using a self-expandable metal stent. In this way, the rectal stump is connected to the bowel, ensuring the patency of the gastrointestinal tract. The third stage takes place after another 4 weeks. Following the radiological confirmation of the anastomosis integrity, the colostomy is transected, closed with a linear stapler, and returned to the abdominal cavity, which avoids an extensive incision and adhesiolysis. The metal stent can be removed or left in place.

A 76-year-old female patient presented to the hospital in an emergency setting due to large bowel perforation secondary to sigmoid diverticulitis. The patient underwent the first stage of the procedure—a primary Hartmann’s resection with a minor modification: the descending colon was mobilized and the remaining sigmoid colon was fixated laterally to the closed rectal stump using four sutures. Three months later the patient was qualified for the subsequent steps of the EndoHARP procedure. Contrast medium was administered through the stoma, followed by the transrectal introduction of an echoendoscope to locate the fixated bowel loop under EUS imaging. Utilizing a delivery system equipped with electrocautery, a self-expandable metal stent was deployed transmurally, connecting the rectal stump to the bowel loop. Four weeks later, a radiological examination was performed, confirming the complete integrity and patency of the anastomosis. The final stage was then initiated: the colostomy was closed using a linear stapler and returned to the abdominal cavity. The patient experienced no intra- or post-procedural complications. At the 2-month follow-up, the patient remained in good clinical condition, and the restored gastrointestinal continuity was functioning properly ( Video 1 ).

The application of the EndoHARP procedure eliminates the need for peritoneal adhesiolysis and traditional suturing of the bowel anastomosis, effectively minimizing the risk of complications associated with a burdensome surgical operation. It is an effective endoluminal technique, potentially increasing the rate of patients in whom permanent stoma reversal is possible.

The EndoHARP procedure. EndoHARP, Endoscopic Ultrasound-guided Hartmann Reversal Procedure.Video 1

Endoscopy_UCTN_Code_TTT_1AS_2AZ

Endoscopy_UCTN_Code_TTT_1AQ_2AF
